# Maternal assessments of family climate in mother-child dyads: investigating the role of maternal borderline personality disorder in mental representations

**DOI:** 10.1186/s40479-025-00306-2

**Published:** 2025-09-08

**Authors:** Anne Jung, Robert Kumsta, Babette Renneberg, Silvia Schneider, Nina Heinrichs

**Affiliations:** 1 Department of Psychology, Clinical Child and Adolescent Psychology and Psychotherapy, Bielefeld University, Bielefeld, Germany; 2 Department of Psychology, Clinical Psychology and Psychotherapy, University of Bremen, Bremen, Germany; 3 Department of Behavioral and Cognitive Sciences, Faculty of Humanities, Education and Social Sciences, University of Luxemburg, Esch-sur-Alzette, Luxemburg; 4 German Center for Mental Health (DZPG), partner site Bochum/Marburg, Bochum, Germany; 5 Department of Education and Psychology, Clinical Psychology and Psychotherapy, Freie Universität Berlin, Berlin, Germany; 6 Clinical Child and Adolescent Psychology, Forschungs- und Behandlungszentrum für psychische Gesundheit, Ruhr-Universität Bochum, Bochum, Germany

**Keywords:** Five minute speech sample, Mental representation, Expressed emotion, Coherence, Attribution, Borderline personality disorder

## Abstract

**Background:**

Family climate substantially influences children’s socio-emotional development. We examined mothers’ mental representations of their children and their relationships in three groups of mothers with young children (0–6 years): mothers (1) with a borderline personality disorder (BPD) (2), with a depressive or anxiety disorder but no BPD (AD/D), or (3) without a current mental disorder (CON). We expected both clinical groups to show more negative mental representations – more expressed emotion reflecting a critical attitude toward the child in general, more hostile attributions to child misbehavior in particular, and a less balanced view of the child (i.e., lower narrative coherence) – than CON mothers. We also expected mothers with BPD to have more impaired mental representations than mothers with AD/D.

**Methods:**

Data were collected as part of an intervention study (at the pre-intervention assessment). To assess parental attributions, 172 mothers with BPD, 69 mothers with AD/D, and 96 CON mothers provided responses to vignettes and participated in a five-minute speech sample coded for expressed emotion and narrative coherence.

**Results:**

BPD was associated with more criticism (OR = 3.17 and OR = 3.93) in comparison with CON mothers and mothers with AD/D, and with lower narrative coherence (OR = 5.45) compared with CON mothers but not compared with mothers with AD/D (OR = 1.41). Only narrative coherence remained significantly associated with group membership after education was controlled for. Mothers with BPD also showed more hostile attributions than CON mothers, with the AD/D group in between.

**Conclusion:**

Without controlling for maternal education, critical attitudes toward the child in general were specifically associated with BPD, hostile attributions were less clearly associated, and narrative coherence was transdiagnostically associated with mental disorders in general. Once education was controlled for, disorder-specific associations were no longer observed, while transdiagnostic associations were maintained. Early interventions may specifically aim to decrease levels of criticism, help mothers increase non-hostile attributions of child misbehavior, and support mothers in building more coherent mental representations of their children.

**Trial registration:**

This study was pre-registered at the German Registry of Clinical Studies (DRKS-ID: DRKS00020460).

**Supplementary Information:**

The online version contains supplementary material available at 10.1186/s40479-025-00306-2.

## Theoretical background

How caregivers think about and relate to their children – i.e., how they mentally represent their children, their children’s behavior, and their relationships with their children – influences how caregivers actually interact with their offspring [1], thereby contributing to the family climate in which the children grow up and develop [2]. Family climate encompasses all “processes operating within the family” [3], such as parent-child interactions, family satisfaction, and the levels of positive or negative affect that are displayed [3, 4].

The assumption that parental mental representations and parent-child interactions are connected is a fundamental tenet of attachment theory [5, 6] as well as of social learning theory, where links between parental attributions and parenting are a core assumption [7, 8]. Caregiver-child experiences within the family climate have a substantial impact on children’s further development [9]. The climate within a family is shaped not only by the parents’ characteristics (e.g., cognitions, attitudes, parenting goals, caregiving, and parenting) [4] but also by the children’s characteristics (e.g., how the children exhibit compliance, control, or affection, or by how the children organize their own feelings or cognitions) [2], which, in turn, influence their parents’ reactions and vice versa [10, 11]. Characteristics of parents and children are reciprocal and transactional in nature, and parents and children mutually affect each other’s behaviors, cognitions, and emotions [11].

The family climate is moreover influenced by a variety of factors that affect both the caregiver and the child, such as the mental health of the parent or child, socio-economic status, or the parent’s level of education [12, 13]. Mental disorders can have an effect on how parents process and express emotions [14–16], thereby shaping the family’s climate through the parents’ mental representations of their children and their relationships. The ways in which parents think about and relate to their children have been conceptualized as potential mechanisms by which mental health problems are transmitted from parent to child [17, 18]. Thus, parental mental disorders are often conceptualized as a risk factor for a variety of child outcomes [17].

The present article focuses on the role that caregivers’ characteristics play in determining the family climate, with a particular emphasis on mental representations and attributions in the presence of parental mental disorders in general and borderline personality disorder (BPD) in particular.

BPD is characterized by difficulties in emotion regulation, affective instability, impaired executive functioning, and fears of abandonment, all of which can also be reflected in dysfunctional relationship behavior [19]. Being affected by BPD is thus likely to also spill-over into the parent-child relationship [20] and may pose a challenge to creating a healthy family climate in which a parent and child are comfortable with each other. When focusing on self-reported outcomes, parental BPD is associated with less empathy and more maladaptive parenting styles [21] as well as an increase in the potential for child abuse [22]. Systematic reviews that have relied on questionnaires, and to some extent also on behavioral observations, have reported similar results: Parental BPD is associated with less sensitivity and engagement, more overprotectiveness and hostility, and a higher risk of inducing role reversals compared with parents without a mental disorder [23, 24]. However, there has been no report on the size of such an effect in the reviews. A second limitation of these reviews is related to a major reliance on self-reports: Parents with BPD may have a negativity bias toward their children’s emotional expressions (i.e., a tendency to perceive facial expressions in an overly negative way) that can lead, for example, to viewing the child as being more angry compared with parents with low BPD symptoms [24]. Children of parents with BPD are also at a higher risk for child maltreatment experiences and neglect [25]. It is assumed that the family climate may be an important mediator that links parental mental disorders and child socio-developmental outcomes.

As the majority of primary caregivers are mothers (and not fathers) [26], we decided to focus exclusively on mothers and to examine the role of maternal BPD in mothers’ mental representations of their children. This representation is an internal image that mirrors the family climate while also contributing to it. To our knowledge, no study to date has comprehensively examined how maternal characteristics contribute to family climate in mothers with BPD, with an emphasis on characteristics of the mothers’ general mental representations of their children and the mother-child relationship, as well as with an emphasis on the mothers’ specific representations of child misbehavior. Additionally, and to the best of our knowledge, studies have yet to explore whether impairments in both types of mental representations (general or specific to child misbehavior in particular) are specific to parents with BPD or if they are also present in other mental disorders. For example, Dittrich et al. (2018) identified a heightened potential for abuse not only in mothers with BPD but also in mothers with depression. To shed light on potential associations between mental disorders and maternal mental representations, we compared mothers with BPD with mothers with anxiety or depressive disorders or both (clinical control group) and mothers without a current mental disorder (non-clinical control group). By including two control groups, we could test whether possible associations between BPD and mental representations are specific to the disorder itself (disorder-specific hypothesis) or are instead a general phenomenon of a mental disorder (transdiagnostic hypothesis). We examined different maternal characteristics of mental representations as indicators of and contributing to family climate, namely, expressed emotion, narrative coherence, and parental attribution.

### Expressed emotion (EE)

Expressed emotion [27] is an important indicator of family climate that may reflect a parent’s general representation of their child and their relationship with their child [28]. EE is an indicator of a person’s affective attitude to a close relative, focusing on criticism and emotional overinvolvement [17, 29, 30] and is coded as present (i.e., high EE) or absent (i.e., low EE). High EE is assumed to reflect disruptions and disturbances in the dyad’s emotional climate, even when it is measured on the individual level [17, 30]. Expressed emotion is either deduced from what is said (content) or how it is said (emotional intensity) [27, 31]. Content typically refers to displays of criticism, such as negative statements, expressions of disapproval, or a negative tone of voice [28], sometimes also to displays of hostility [28], as indicators of a negative family climate (referred to as high expressed emotion). In some articles, warmth is additionally coded as a reflection of low expressed emotion [32], while in others, it is not considered in the overall assessment [28]. Intensity, meanwhile, is deduced from indicators of emotional overinvolvement, for example, self-sacrifice, overconcern, parental exaggeration, enmeshment, or overloaded emotional displays such as crying during the assessment [30, 31]. Higher EE is associated with parents of children who are affected by a mental disorder, compared with parents of children without a mental disorder [33, 34]. Parental EE might therefore also exacerbate the child’s risk of developing a mental disorder.

Verbal affective content is assumed to reflect the emotional valence and, additionally, relevant behaviors and parent-child interaction processes expressed in daily family situations [32]. Significant associations between EE scores – mostly for criticism as the “core dimension” of EE – and directly observed interactions have consistently been identified, especially for mother-child interactions [28, 35, 36]. A meta-analytic review demonstrated that maternal criticism is significantly related to both internalizing and externalizing symptoms of their offspring, while maternal overinvolvement is connected only to youths’ internalizing symptoms [18]. Criticism in particular is perceived as a key mechanism in the transmission of mental disorders through disrupted parent-child interactions [17].

Given the fact that maternal stress increases a parent’s probability of being classified as critical or overinvolved [37], it seems plausible that the presence of a maternal mental disorder in general might lead to more dysfunctional emotional valence in the family relationship (transdiagnostic hypothesis) or that more dysfunctional emotional valence might specifically be linked to the presence of maternal BPD (disorder-specific hypothesis). To our knowledge, no previous studies have explored the family environments that mothers with BPD create on the basis of maternal expressed emotion. Mothers with BPD are described as more hostile and having a higher risk of inducing role reversals [23, 24]. Interactions with the child are characterized by under-involved on the one hand and over-involved on the other [23], and their children describe them as overinvolved [38]. However, mothers with a history of depression have also shown higher levels of criticism and overinvolvement, whereas current depressive symptoms were also correlated with critical remarks [39–41]. Both parental depression and anxiety disorders are significantly associated with higher criticism, as demonstrated by a meta-analytic review, and criticism can be seen as a specific reaction style of parents with affective mental disorders [17]. A first aim of the present study is to clarify whether this reaction style is truly (disorder-)specific or whether this phenomenon cuts across different mental disorders.

While the construct of EE may reflect the content of a parent’s mental representation (i.e., whether the child and the relationship with the child are represented in a positive and loving way or in a negative and critical way), it does not give a lot of information about the parent’s cognitive “framework” [42]. This information is better integrated in the construct of narrative coherence [43].

### Narrative coherence (NC)

Since humans are creatures that strive for meaningfulness and predictability, they extract meaning from daily life events and aim to remember them coherently – a process that is referred to as “affective meaning making processes” [44]. In the context of parenting and with reference to attachment theory, such affective meaning making processes are considered to have an impact on internal representations and to be impacted by the internal representations in return [44]. A parent’s narrative coherence is defined as their ability to provide a consistent, complex, balanced, and accepting description of their child [44–46]. Coherent descriptions should thereby include the strengths and weaknesses of the child [47]. Being able to produce a coherent narrative is believed to reflect flexible information processing regarding the child and the parent-child relationship [16, 45]. Incoherent narratives may otherwise reflect defensive and dysfunctional information processing mechanisms and may facilitate overwhelmed, intrusive, or withdrawn dyadic behavior [48]. Narrative coherence has its origins in attachment theory [43, 46].

Internal representations influence the interpretation of children’s behavior and have a corresponding effect on the dyadic behavior that is displayed [45, 49]. It is assumed that coherent representations and flexible information processing facilitate accurate interpretations of as well as appropriate reactions to the child’s signals and therefore result in more sensitive caregiving [46, 50]. This assumption is also supported by research findings: Mothers who are able to produce a coherent description of their child are observed as being more emotionally available [47]. Relying on self-report measurements, parents who are classified as coherent also report significantly more family cohesion, less hostility in their own parenting, and more prosocial behavior in their children [51]. Similar to EE, coherence is not only associated with observable behavior in the mothers but also with developmental outcomes in the children. In line with these associations, a mother’s ability to present a coherent portrayal of her child is associated with fewer behavioral problems in the child (i.e., less internalizing and externalizing behavior) [31, 48, 52].

Little is known about narrative coherence in parents who are affected by a mental disorder in general or BPD in particular. We are aware of only one study that investigated narrative coherence and mental disorders, but it did not include parents with a mental disorder themselves and instead included parents of children with a mental disorder. Mothers of these children produced less coherent narratives and described their relationships as less joyous [53]. Related constructs, such as insightfulness, which also originated from attachment theory (for a definition and differentiation of relevant constructs, see Zeegers et al. [54]), were identified as influenced by maternal depression: Mothers with depression provided less complex, less focused, and less coherent answers in insightfulness assessments [55, 56]. Narrative coherence is rooted in the construct of insightfulness and neither has been studied in BPD.

### EE and NC

EE and NC can be derived from the same data source, namely, from parental narratives about their children. Both are indicators of parental mental representations that contribute to and reflect the family climate, but they stem from different theoretical backgrounds and emphasize different aspects of the narratives [16]. The EE construct reflects the family environment and originally focused on attributions made by relatives of patients with schizophrenia [28, 57]. Thus, EE is theoretically centered on relational attributions [28]. Meanwhile, the focus of NC is on the internal processes that facilitate sensitivity [46, 58]. This focus is achieved by exploring the organization of parental thought and speech as a reflection of their own attachment representations that, in turn, correspond to sensitive dyadic behavior [54]. In accordance with these theoretical considerations, EE emphasizes the narrative’s content (and captures relational attributions, i.e., “*what*”), while NC focuses on the organization of the narrative in terms of its coherence (and captures information processing and storage and retrieval processes of the aforementioned relational attributions, i.e., “*how*”) [58, 59].

The developer of the NC coding system found significant negative correlations (−0.21 and − 0.22, respectively) between negative remarks in the EE ratings and coherence [48, 59], although this pattern was not always found [31], thereby indicating rather small overlap in constructs. Other components of EE, such as the number of positive remarks, were not related to NC at all [48]. NC contributed uniquely to the amount of variance that could be explained in child development [31] and to the children’s own portrayal of the mother-child relationship as responsive and supportive [59]. These initial studies suggest that NC and EE capture different constructs of parental mental representations while not being completely independent of each other [31]. Thus, high expressed emotion and incoherence may both reflect difficulties in the parent-child relationship with implications for child well-being, but they make different contributions [16]. While EE is associated with poorer socio-emotional adjustment in children and disruptive behavior, parental representations of the child are associated with a child’s positive representation of the parent [16]. However, evidence is limited to a small number of studies, none focusing specifically on mothers with different mental disorders. The extent to which it is beneficial to simultaneously examine constructs from different theoretical backgrounds as indicators of family climate has not yet been sufficiently investigated. As the two constructs focus on different aspects of the narrative, they may be uniquely associated with the presence of a mental disorder. Alternatively, they might not be uniquely associated with the presence of a mental disorder, as indicated by strong correlations with each other, maternal psychopathology, or both. This information would be useful for researchers and clinicians, as it would allow them to select the construct that best suits their needs for assessing impairments in mental disorders or for guiding interventions. A deeper understanding of whether and how mental disorders influence different aspects of mental representations may also be helpful for tailoring parenting training programs. Using the same data source to assess both constructs (i.e., the same narrative) allows constructs derived from different theories to be examined and has the advantage that any potential gains from including different constructs cannot be attributed to methodological differences (i.e., reducing variance between the outcomes of the two constructs due to methodological differences).

### Parental attributions of child misbehavior

While EE and NC reflect mental representations of the child and the relationship in general, social learning theory also emphasizes the importance of how parents think about child misbehavior [7, 8], which may specifically reflect parental mental representations of child misbehavior. Parental causal interpretations of aversive child behavior as “internal, stable, and global to the child” [60] are a well-researched risk factor for child maltreatment [61]. Such negative interpretations are viewed as key drivers in coercive, dysfunctional behaviors from parents toward children by facilitating more hostile, aggressive responses (i.e., eliciting more negative affect in parents and more hostile behavior toward the child), and, as such, may also contribute to the emotional climate within a family. Attributions can therefore be seen as the cognitive predisposition to express criticism and hostility directly. Azar and colleagues refer to parental attributions as the “products of cognitive activity ‘in the moment’ (the judgements resulting from activation of schema and problem solving)” [62] that may be prone to bias (i.e., attributing negative behavior to the child even if not justified). In contrast to the construct of EE, where the child and the mother-child relationship in general are reflected in the mother’s mental representations, we propose that parental attributions of child misbehavior may also be subsumed under the umbrella of mental representations but with a specific focus on representations regarding aversive child behavior.

These parental attributions of child misbehavior link (limited) executive functioning capacities (e.g., “capacity to shift set as new information is available” [62]) to parental responses (e.g., harsh parenting behavior, lack of monitoring or support) in the Social Information Processing model [63]. The model posits a link between parental and contextual characteristics and an increased risk of child maltreatment, based on cognitive-behavioral theories [63]. Based on social learning theory, the model provides a theoretical framework for how mental representations or cognitive schemas are related to observable dyadic behavior and thus contribute to family climate. It proposes that parental reactions (e.g., harsh physical discipline) to aversive child behavior (e.g., the parent is busy, but the child wants something) are guided by contextual resources (e.g., poverty), child characteristics (e.g., age), and caregiver characteristics, such as parental expectations of the child (cognitive schema), the parent’s ability to take the child’s perspective (executive functioning capacity), and the parent’s interpretation of the child’s behavior (attribution) [63].

Being a parent with BPD may be associated with problems in social information processing, as individuals with BPD are assumed to struggle to distinguish between their own and others’ intentions and are prone to misinterpreting their child’s behavior [64]. Mothers with depression are, however, also known to have a tendency to interpret their child’s problem behavior as intentional as well as controllable [65] and are therefore likely to resort to “child-blaming” attributions [39]. Thus, it is again unclear how specific these processes are to a given mental disorder.

### The current study

To our knowledge, most studies of mothers with BPD to date have primarily examined only single aspects of mental representations, and thus family climate, by looking at either EE (but mostly from the perspective of adult relatives of patients with BPD [66, 67]) or parenting behavior (mostly relying on self-reports [25, 68]) or focusing on child personality psychopathology and social information processing without linking these aspects to a relational mother-child framework (e.g., by investigating possible links between personality disorder clusters in adolescents and attributions of conflict situations with peers [69]). Most importantly, the majority of studies have compared mothers with BPD with a group of mothers not affected by a mental disorder. The current evidence does not allow conclusions to be drawn about specificity in disorders or about different aspects of mental representations. With the current study, we aim to investigate different aspects of mental representations and, thus, aspects of family climate (assessed via EE, NC, and parental attributions) in mothers with BPD compared with a clinical comparison group, comprising mothers with anxiety disorders, depression, or both (AD/D), as well as a healthy comparison group comprising mothers without a current mental disorder (CON). Anxiety and depressive disorders were chosen as the clinical comparisons, as these disorders have high prevalence rates [70], affect parents and their children frequently, and are thereby particularly relevant. We hypothesized that the type of mental disorder would predict the aforementioned aspects of mental representations and that mothers in either clinical group (BPD or AD/D) would produce less coherent speech samples, would score higher on criticism and/or emotional overinvolvement (i.e., higher EE), and would show more negative parental attributions of the child’s intentions than mothers without a mental disorder (transdiagnostic hypothesis) [17, 71]. Additionally, we hypothesized that BPD would be associated with more impaired mental representations than AD/D would. Thus, we assumed a disorder-specific association in that the type of mental disorder would lead to distinct patterns in terms of narrative coherence, EE, and parental attributions.

## Methods

This study is part of “ProChild”, a multicentered study in Germany consisting of five subprojects aimed at evaluating the efficacy of an outpatient parenting program for mothers with BPD (subproject 1) while examining child development (subproject 2), family climate (subproject 3), and epigenetic mechanisms (subproject 4). Additionally, the collaboration between childcare institutions, psychotherapists, and mothers with BPD are being investigated (subproject 5). The present article covers the main cross-sectional research questions from subproject 3 and specifically concentrates on mothers’ mental representations, while the behavioral aspects of family climate in terms of dyadic behavior (either parent-perceived or observer-rated) will be reported separately (in preparation). The project was funded by the German Federal Ministry of Education and Research (funding ID: 01KR1805C), and ethical approval was obtained from the ethics committee of the DGPs (No. RennebergBabette2019-07-29VADM). The primary hypotheses from subproject 3 were preregistered in the German Clinical Trial Register on February 02, 2020 (ID: DRKS00020460); a study protocol for subproject 1, which also provides an overview of the general assessments, was published in 2022 [72].

### Procedure

Data were collected (2019–2024) in four German cities: Berlin, Bochum, Bremen, and Bielefeld (Bielefeld replaced Bremen from 2023 on). Three groups of mothers with children between 6 months and 6 years of age were recruited via psychotherapists, clinics, youth welfare organizations, kindergartens, and social media. These mothers had to meet the criteria for a specific mental disorder (for either clinical group) or none at all (for the non-clinical control group): We included (1) mothers with a borderline personality disorder (BPD), (2) mothers with anxiety disorder, depression, or both (AD/D), and (3) mothers without any mental health problems within the last seven years (CON) as well as their children. All interested mothers were first screened by phone to check eligibility and further inclusion (i.e., regular, at least weekly, contact with their child, adequate German language skills to participate in the assessment; for the BPD group: mothers had to have either completed BPD-related treatment or currently be in treatment) and exclusion criteria (i.e., acute risk of child maltreatment, current suicidality, acute alcohol or drug dependence, acute psychotic symptoms, or a diagnosis of mental retardation in the mother). A lifetime diagnosis of BPD was an exclusion criterion for both control groups (AD/D, CON); and any other mental disorder within the last 7 years – during their child’s lifetime – was an exclusion criterion for CON mothers. Children could not be blind, deaf, or severely limited in mobility. Two visits were scheduled if eligibility was met from the screening: the first aimed at confirming group membership with the Structural Clinical Interview for DSM-5 disorders [73, 74] (duration varied between 1 and 3 h based on the number of diagnoses), providing informed consent, and conducting a Five Minute Speech Sample (FMSS) [27]. If eligibility was confirmed, mother-child interaction assessments were conducted during the second visit, and questionnaires were provided to be completed between these appointments. Mothers received financial compensation for both visits (50 Euro in total), and children received a present at the end (e.g., Pixie book or bubbles). The project design included one assessment for each group (“baseline”; before the intervention), followed by second (“post”; after intervention) and third (“follow-up”; after 6 months) assessments for the BPD group. The CON group was also assessed again at post and follow-up. The present data are from the first assessment from subproject 3. A full list of instruments related to subproject 3 is provided in the OSF repository.

### Participants

In total, 351 mothers and their children participated in the ProChild study. For the present research questions, we had data for *n* = 337 dyads with at least one of the outcome measures available (due to, e.g., technical difficulties with recording the FMSS or invalid responses to the Child Vignettes; see Table 1). Across all three groups, the mothers were 34 years old on average (5.93 SD), and the children were mostly toddlers with an average age of 3 years (1.84 SD).

**Table 1:** Sample descriptives

	BPD	AD/D	CON	Group comparison
n	172	69	96	
*Mothers*				
Age: mean (SD)	32.95 (6.21)	34.43 (5.53)	34.72 (5.51)	*F*(2, 333) = 3.4, *p* =.037
Education low: n (%)	98 (57%)	19 (28%)	10 (10%)	Kruskal Wallis$\:\chi\:$²(2) = 60.5, *p* <.001
*Children*				
Age: mean (SD)	3.43 (1.91)	3.30 (1.77)	2.81 (1.71)	*F*(2, 334) = 3.54, *p* =.030
Girls: n (%)	88 (51%)	37 (54%)	55 (57%)	$\:\chi\:$²(2) = 0.93, *p* =.628
Clinical diagnosis: n (%)	57 (33%)	27 (39%)	13 (14%)	$\:\chi\:$²(2) = 16.5, *p* <.001
*Dyad*				
Contact with youth welfare system: n (%)	103 (60%)	25 (36%)	13 (14%)	$\:\chi\:$²(2) = 52.96, *p* <.001
Children in foster care (lifetime): n (%)	10 (6%)	2 (3%)	1 (1%)	$\:\chi\:$²(2) = 3.91, *p* =.141

*BPD *Borderline personality disorder group, *AD/D *Anxiety/depressive disorder group, *CON *Control group without a current mental disorder. Child age is displayed in years. Child clinical diagnosis: 2 missing values, contact with youth welfare system: 7 missing values, foster care: 8 missing values. All subsequent analyses were also calculated with mothers with children in foster care (past or present) excluded (see Additional file 1).

### Measures

#### Structural clinical interview for DSM-5 disorders (SCID-5)

Maternal mental disorders were assessed with an adapted German version of the Structural Clinical Interview for DSM-5 Disorders (SCID-5) with the Clinical Version [74] and the Personality Disorders Version [73]. The following sections were used: affective disorders, psychotic symptoms, substance dependence, panic disorder, agoraphobia, social anxiety disorder, general anxiety disorders, posttraumatic stress disorder, and borderline personality disorder; other sections were not relevant to the project and therefore not included. BPD was assessed as a lifetime diagnosis; all of the other diagnoses were assessed as current diagnoses. In the AD/D group, mothers with remitted symptoms were also included as long as they fulfilled all the criteria of an anxiety or depressive disorder within the last two months. Raters received initial training and ongoing supervision, which included feedback sessions based on reviewed interviews. When discrepancies or issues in quality were identified, corrective feedback was provided, and the diagnosis was adjusted. All interviews were video-recorded, and a subset (20%) was independently reviewed by a senior clinician with expertise in SCID administration. No interviews were deemed low quality (e.g., significant deviation from protocol, incomplete responses, or leading questions), so that no re-administration by another trained rater was necessary. Overall, the majority of interviews met the aforementioned quality standards.

#### Five minute speech sample (FMSS)

At their first appointment, mothers were asked to talk about their child and their relationship with their child for five minutes [27]. These narratives can be used, for instance, to gain insight into the emotion displayed by a parent toward their child and is thereby assumed to reflect processes typical of the interaction between them [36, 44]. Originally, the FMSS was developed as an alternative to the Camberwell Family Interview to identify conditions that increased the likelihood of relapse – namely, EE [16, 27, 30]. Since its development, the FMSS has been used in other contexts, such as to assess the family climate in parent-child dyads [16, 36] and can provide valid insights into both expressed emotion and narrative coherence [16, 27]. There is considerable evidence that the FMSS can be used as a reflection of parent-child relationship quality and parent-child dynamics in Western cultures [16] via its robust associations with observational parental dyadic behavior [36].

The FMSS was administered directly after we obtained written informed consent and before we administered the SCID-5. The instructions followed the recommendations in the literature [27]: “I’d like to hear your thoughts about [child’s name], in your own words and without me interrupting you with any questions or comments. When I ask you to begin, I’d like you to speak for 5 min, telling me what kind of person [child’s name] is and how you get along together. After you have begun to speak, I prefer not to answer any questions. Are there any questions you would like to ask me before we begin?”

All speech samples were audiotaped and transcribed before coding. Coders were blind to assessment point, study site, and group membership. The EE and NC coding were done by separate coder teams, each consisting of three psychology students who participated in a training process to establish inter-coder reliability prior to coding the study tapes. The training processes were administered by researchers who were trained and reliable themselves (the first and last authors of this manuscript) and were based on the corresponding coding manuals (see below).

#### Expressed emotion (EE)

Expressed emotion was coded in line with the German translation of the original coding manual [27, 75], which includes the original EE rating as well as an additional variant that also includes covert criticism [76]. Each speech sample was coded for relationship quality (positive, neutral, negative), criticism (CRIT), and emotional overinvolvement (EOI). Positive *relationship quality* was rated if the mother expressed interest in her child (e.g., described joint activities) or if the relationship was described as loving and positive. A neutral relationship was coded if both positive and negative relationship statements were provided or if no information was given about the nature of the relationship. A negative relationship was coded in the presence of clear negative relationship statements (e.g., “We can’t have a conversation without it ending in an argument”). Critical remarks were coded once on the basis of content (overt and covert) and once on the basis of tone. *Overt criticism* was coded if the mother clearly rejected certain behaviors or characteristics of the child (e.g., “I don’t like.”). It was also coded in the case of excessive details/embellishment (e.g., an exaggerated description of unloved behaviors or characteristics). *Covert criticism*, by contrast, included statements in which a mother mentioned characteristics or behaviors of her child that could be perceived as annoying (e.g., “She is too ambitious”; “He is very fussy”) or mentioned negative statements by third parties without clear differentiation. *Critical tone of voice* was coded on the basis of changes in pitch or voice modulation, so that even statements that were not critical in themselves could lead to an overall assessment as a critical statement on the basis of a critical tone of voice. *Emotional overinvolvement* (EOI) was assessed using two subscales: self-sacrificing/overprotection and excessive emotional display. *Overprotection* was coded for behaviors in which mothers extremely neglected or sacrificed their own needs (e.g., “I would like to go on vacation, but I don’t dare, just in case she needs something”) or showed a clear lack of objectivity. Crying or the inability to continue speaking due to clear affective involvement was coded as *excessive emotional display*.

Similarly to previous research [34, 37], EOI was rarely coded. Therefore, we computed an overall dichotomized status where each sample was coded as low (LEE) or high EE (HEE). We coded each sample twice: once according to the original manual [27, 76] and once with the additional coding of covert criticism [75]. Accordingly, there were two EE scores per mother included in the analyses: EE score 1 (EE1; overt criticism) and EE score 2 (EE2; covert criticism). HEE was coded when any of the following criteria were met: (a) negative relationship quality, (b) overt criticism or critical tone of voice (according to the original manual; EE score 1) and/or covert criticism (according to the additional coding; EE score 2), or (c) EOI [27, 75, 76].

Reliability in EE was measured before any study tapes were coded (EE1: 87–93%, Holley and Guilford’s *G* 0.73–0.87; EE2: 73–80%, *G* 0.47–0.60; reliability of each coder compared with experts’ codes for 15 narratives). Due to the low base rate of observations and the known sensitivity of Cohen’s Kappa in these cases, Holley and Guilford’s *G* was used to evaluate interrater reliability [77]. To prevent coder shift, reliability was checked during the main coding procedure, with 30 study tapes (roughly 5%) that were triple coded, separately by each coder. Reliability was checked about halfway through and toward the end of the coding (EE1: 87–97%, *G* 0.73–0.93, EE2: 70–90%, *G* 0.40 − 0.80; reliability between coders).

EE coding as a measure of mother-child relationships and family climate – particularly the coding of criticism – is robustly associated with observable dyadic parental behavior [36, 78], underlining its external validity. No evidence from behavioral observations supports the robustness of EOI [36]. An adaptation of the original EE coding manual, the Preschool FMSS, which also includes an operationalization of critical remarks similar to Magaña’s definition, showed good code-recode reliability as well as moderate test-retest reliability [34].

#### Narrative coherence (NC)

Each speech sample was additionally coded for NC [43] on six scales (focus, i.e., shifting to irrelevant topics; elaboration, i.e., details/richness of the narratives; separateness with boundary dissolution, i.e., seeing the child as an independent person; concern/worry; acceptance/rejection; and complexity, i.e., multifaceted child portrayal) that provided a global narrative coherence score. This score represents an integration of the six dimensions and reflects the overall organization and consistency of the mother’s narrative, including an overall positive description of the child. Each scale was evaluated on a 7-point Likert scale ranging from 1 to 7. Lower scores between 1 and 4 reflect an incoherent narrative (i.e., FMSS is one-sided, meager, or contradictory), whereas higher scores between 5 and 7 represent a coherent FMSS (i.e., consistent, complex, and elaborated narrative) [47]. We used the dichotomized coding (coherent vs. incoherent) in our analyses.

The reliability prior to coding the study tapes was acceptable (67–93%, *G* 0.33–0.87; reliability of each coder compared with experts’ codes for 15 narratives). Again, we checked the reliability by triple rating 28 study tapes in total (roughly 5% of all transcripts), about halfway through and toward the end of coding (68–75%, *G* 0.36–0.50; reliability between coders).

The external validity of the NC coding was established for associations between coherent transcripts and fewer behavioral problems in children [48], more positive mental representations of the children themselves [59], and in predicting changes in the adjustment of children with self-regulation difficulties [79]. To our knowledge, other psychometric properties have not yet been investigated.

### Child vignettes (CV)

Parental attributions of child misbehavior were assessed with Child Vignettes [62, 80], consisting of 18 hypothetical vignettes that described aversive child behavior with varying degrees of intentionality. The scenarios were differentiated according to whether the child’s behavior could be considered clearly intentional (e.g., misbehaving as a direct consequence of disciplinary action), clearly unintentional (e.g., a lack of developmental capability in specific areas), or ambiguous (e.g., a situation with insufficient information available), with six scenarios in each category. Mothers were requested to imagine that the child depicted in the scenario was their own and were then required to rate (1) how much the child engaged in the behavior to annoy them (intentionality), (2) the extent to which they would punish the child’s behavior (punishment), and (3) how much they perceived the behavior to be caused by their own behavior (cause), using a 9-point Likert scale (1 “not at all” to 9 “very much”). For each type of scenario, sum scores for intentionality, punishment, and cause were computed separately (possible range 6–54) as well as a total score across all types of scenarios, although here, we examined only the intentionality scale. Following S. Azar’s recommendation, we used the intentionality scale for ambiguous scenarios only to check for attributional biases in the mothers (personal communication S. Azar, 11/2024), with higher scores indicating more hostile attributions.

The Child Vignettes have previously been found to have good internal consistency (Cronbach’s alpha = 0.90) for intentionality, and this score differentiates maltreating from non-abusive mothers by predicting child maltreatment [62, 80, 81]. Cronbach’s alpha for the current study was 0.75.

### Statistical analysis

An a priori power analysis was calculated for the entire consortium to determine the required sample size. We performed regression analyses in R Studio, version 4.4.2 [82], using the *lm.beta* [83] and *QuantPsyc* packages [84]. Expressed emotion (LEE vs. HEE) and narrative coherence (coherent vs. incoherent) were analyzed as dichotomous outcome variables, so we computed separate logistic regressions. Each regression analysis was computed with group as a predictor, which we contrasted beforehand (i.e., BPD vs. AD/D, BPD vs. CON). Relying on logistic regression and the group variable as a contrast prevented us from comparing the clinical control group with the healthy control group. Hence, some assumptions could not be tested if we wanted to control for maternal education. However, the main research questions (BPD vs. clinical control group and BPD vs. healthy control group) could be tested this way, including maternal education as an already well-known characteristic that also drives FMSS results. The logistic regression allowed us to control for significant differences between groups – such as maternal education – so we ran each model again with maternal education (low vs. high) as another predictor. Mothers with an educational qualification from secondary school or higher (Gymnasium and Abitur, comparable to a high school diploma or A-levels) were categorized as having a higher educational qualification, while mothers with educational qualifications under this level were categorized as having a lower educational qualification. As the inclusion of more control variables (e.g., age of mother and child) would have complicated the model, we refrained from adding these variables to the analyses. All assumptions for the logistic regressions were tested beforehand (see Additional file 1).

For hostile attributions as an outcome, we used the sum score of the intentionality scale of ambiguous scenarios and planned to conduct a linear regression. However, since many conditions for a linear regression were not met (see Additional file 1), we switched to the Kruskal-Wallis test. This method enabled us to compare all three groups while still using the whole range of the interval-scaled outcome parental attribution. In addition, we performed the Jonckheere-Terpstra test to investigate the assumed trend in our data (BPD with highest hostile attributions score, followed by AD/D, followed by CON). We were not able to include maternal education as a covariate in this model and therefore could not examine its influence on parental attributions.

## Results

Descriptive statistics for the outcome variables are depicted in Table 2. Inter-correlations between the dependent variables, group, and maternal education were also calculated (see Additional file 1).

**Table 2:** Descriptives for expressed emotion, narrative coherence, and parental attributions (*N* = 337)

	BPD	AD/D	CON
*Overt criticism (EE1)*			
n HEE (%)	26 (16%)	3 (4%)	5 (5%)
n total	167	67	91
*Covert criticism (EE2)*			
n HEE (%)	74 (44%)	22 (33%)	21 (23%)
n total	167	67	91
*Narrative coherence (NC)*			
n incoherent (%)	106 (63%)	37 (55%)	22 (24%)
n total	167	67	91
*Hostile attributions*			
mean (SD)	9.082 (4.315)	7.866 (3.481)	6.906 (1.795)
n total	159	67	96

*N* = 12 missing data in EE, NC, and *n* = 15 in hostile attributions. BPD: borderline personality disorder group, AD/D: anxiety/depressive disorder group, CON: control group without a current mental disorder, NC: Narrative Coherence. Possible range of the Child Vignettes for assessing hostile attributions: 6–54

### Maternal mental disorders and EE

Overall, 16% of mothers with BPD, 4% of mothers with AD/D, and 5% of the CON group expressed high overt criticism toward their child (HEE, see Table 2). It is noteworthy that the majority of mothers across all three groups were classified as low in expressed emotions (i.e., not critical or emotionally overinvolved). Group status was a significant predictor of HEE (see Table 3): The probability of being classified as critical was higher in mothers with BPD than in mothers with AD/D (OR = 3.93) and in those without mental disorders (OR = 3.17). Once maternal education was controlled for, differences between the groups were no longer evident (see Table 3). A lower education level significantly predicted more criticism in mothers (OR = 2.24), regardless of group membership.

**Table 3:** Logistic regression of group (and maternal education) on EE1 score (overt criticism and/or emotional overinvolvement).

	b	SE	z	*p*	Odds Ratio (OR); BPD vs. CON or AD/D	OR 95% CI; BPD vs. CON or AD/D	Odds Ratio (OR); CON or AD/D vs. BPD	OR 95% CI; CON or AD/D vs. BPD
Model without Maternal Education								
Intercept	−1.691	0.21	−7.92	< 0.001***				
Group (BPD vs. CON)	−1.154	0.51	−2.28	0.023*	0.32	[0.104–0.788]	3.17	[1.269–9.65]
Group (BPD vs. AD/D)	−1.37	0.63	−2.18	0.029*	0.25	[0.059–0.756]	3.93	[1.323–16.917]
*Model with Maternal Education*								
Intercept	−2.198	0.36	−6.17	< 0.001***				
Group (BPD vs. CON)	−0.769	0.55	−1.40	0.161	0.46	[0.143–1.281]	2.16	[0.781–6.981]
Group (BPD vs. AD/D)	−1.128	0.64	−1.76	0.079	0.32	[0.074–0.996]	3.09	[1.004–13.524]
Education (high vs. low)	0.805	0.41	1.97	0.049*	2.24	[1.018–5.102]		

*N* = 325 (n BPD: 167, n AD/D: 67, n CON: 91), Model without Maternal Education: R² = 0.047 (Hosmer-Lemeshow), R² = 0.031 (Cox-Snell), R² = 0.063 (Nagelkerke). Model$\:\chi\:$²(2) = 10.15, *p* =.006**, Model with Maternal Education: R² = 0.065 (Hosmer-Lemeshow), R² = 0.043 (Cox-Snell), R² = 0.087 (Nagelkerke). Model$\:\chi\:$²(3) = 14.17, *p* =.003**

When considering covert criticism, rates of high EE increased considerably (roughly 28% more in BPD, 29% more in AD/D, and 18% more in CON, see Table 2). Group status predicted the probability of being rated as HEE – when covert critical remarks were included – for the comparison between mothers with BPD and mothers without any mental disorder but not for comparisons with mothers with AD/D (see Table 4). The probability of being critical was higher in mothers with BPD than in those without a mental disorder (OR = 2.65). This effect remained significant even when maternal education was controlled for (OR = 2.05, see Table 4), though low maternal education also significantly predicted covert criticism, such that mothers with less education were more critical (OR = 1.78).

**Table 4:** Logistic regression of group (and maternal education) on EE2 score (overt criticism, Covert criticism, and/or emotional overinvolvement)

	b	SE	z	*p*	Odds Ratio (OR); BPD vs. CON or AD/D	OR 95% CI; BPD vs. CON or AD/D	Odds Ratio (OR); CON or AD/D vs. BPD	OR 95% CI; CON or AD/D vs. BPD
Model without Maternal Education								
Intercept	−0.229	0.16	−1.47	0.142				
Group (BPD vs. CON)	−0.975	0.29	−3.32	0.001**	0.38	[0.208–0.662]	2.65	[1.511–4.796]
Group (BPD vs. AD/D)	−0.487	0.30	−1.61	0.108	0.61	[0.335–1.104]	1.63	[0.906–2.986]
*Model with Maternal Education*								
Intercept	−0.558	0.22	−2.55	0.011*				
Group (BPD vs. CON)	−0.718	0.32	−2.26	0.024*	0.49	[0.259–0.9]	2.05	[1.111–3.866]
Group (BPD vs. AD/D)	−0.314	0.32	−0.99	0.320	0.73	[0.39–1.348]	1.37	[0.742–2.564]
Education (high vs. low)	0.576	0.26	2.21	0.027*	1.78	[1.067–2.972]		

*N* = 325 (n BPD: 167, n AD/D: 67, n CON: 91), Model without Maternal Education: R² = 0.029 (Hosmer-Lemeshow), R² = 0.037 (Cox-Snell), R² = 0.051 (Nagelkerke). Model$\:\chi\:$²(2) = 12.24, *p* =.002*, Model with Maternal Education: R² = 0.04 (Hosmer-Lemeshow), R² = 0.051 (Cox-Snell), R² = 0.07 (Nagelkerke). Model$\:\chi\:$²(3) = 17.12, *p* =.001**

### Maternal mental disorders and NC

For narrative coherence, 63% (BPD), 55% (AD/D), and 24% (CON) of the mothers were categorized as incoherent (see Table 2). The majority of mothers in both clinical groups (BPD and AD/D) were incoherent, while the majority of mothers without mental disorders described their child in a complex, balanced, and coherent manner. Group status significantly predicted incoherence for mothers with BPD in comparison with those without a mental disorder (OR = 5.45) but not in comparison with M-AD/D (OR = 1.41, see Table 5). Also, when we controlled for maternal education, this effect was still significant (see Table 5). Including maternal education, the odds that mothers with BPD were incoherent decreased (OR = 4.14). Lower educational background predicted a higher risk of incoherence (OR = 1.94).

**Table 5:** Logistic regression of group (and maternal education) on narrative coherence.

	b	SE	z	*p*	Odds Ratio (OR); BPD vs. CON or AD/D	OR 95% CI; BPD vs. CON or AD/D	Odds Ratio (OR); CON or AD/D vs. BPD	OR 95% CI; CON or AD/D vs. BPD
Model without Maternal Education								
Intercept	0.553	0.16	3.44	0.001**				
Group (BPD vs. CON)	−1.696	0.29	−5.79	< 0.001***	0.18	[0.102–0.321]	5.45	[3.112–9.843]
Group (BPD vs. AD/D)	−0.343	0.29	−1.17	0.243	0.71	[0.399–1.266]	1.41	[0.129–0.505]
*Model with Maternal Education*								
Intercept	0.194	0.21	0.91	0.364				
Group (BPD vs. CON)	−1.42	0.31	−4.55	< 0.001***	0.24	[0.129–0.441]	4.14	[2.27–7.735]
Group (BPD vs. AD/D)	−0.146	0.31	−0.47	0.635	0.86	[0.475–1.584]	1.16	[0.631–2.105]
Education (high vs. low)	0.662	0.26	2.50	0.012*	1.94	[1.157–3.269]		

*N* = 325 (n BPD: 167, n AD/D: 67, n CON: 91), Model without Maternal Education: R² = 0.085 (Hosmer-Lemeshow), R² = 0.111 (Cox-Snell), R² = 0.149 (Nagelkerke). Model$\:\chi\:$²(2) = 38.42, *p* <.001***, Model with Maternal Education: R² = 0.099 (Hosmer-Lemeshow), R² = 0.129 (Cox-Snell), R² = 0.171 (Nagelkerke). Model$\:\chi\:$²(3) = 44.73, *p* <.001***

### Maternal mental disorders and attribution

We also investigated whether group status significantly differentiated the level of hostile attributions in ambiguous scenarios. In all groups, the mean scores of hostile attributions were rather low, with individual scores ranging from 6 to 29 (see Table 2 for detailed information per group). The number of hostile attributions differed between groups, *H*(2) = 24.73, *p* <.001. Two-group post hoc comparisons of the mean ranks between the groups revealed a significant difference between the BPD and CON groups in hostile attributions (*difference* = 54.643, *critical difference* = 28.807) but not between the BPD and AD/D groups (*difference* = 26.621, *critical difference* = 32.462) or the AD/D and CON groups (*difference* = 28.021, *critical difference* = 35.480). As expected, Jonckheere-Terpstra’s test showed a significant trend in the data: more hostile attributions in the M-BPD than in the M-AD/D group, which in turn displayed more hostile attributions than mothers in the CON group, *J* = 12,127, *p* <.001.

## Discussion

The present study is the first to comprehensively investigate whether and how mental representations contribute to and reflect the family climate in mothers with BPD and also the first to include mothers with anxiety disorders or depression (AD/D) and mothers without a mental disorder (CON). As expected, BPD was associated with worse family climate across different aspects of mental representations compared with mothers without a mental disorder: Mothers with BPD expressed more criticism (overt and covert), were more incoherent, and perceived more negative child intentions in ambiguous scenarios. The highest Odds Ratio occurred for narrative coherence, indicating that mothers with BPD were roughly five (without controlling for education) or four times (with controlling for education) more likely to describe their child and their relationship incoherently: They gave fewer examples of their child’s behavior, were more generic or imbalanced between positive and negative aspects, and either focused on only negative aspects or idealized their child. However, unexpectedly, these differences were not all maintained when compared with another clinical group: Only overt criticism differed significantly between the BPD and AD/D groups. In fact, the three groups descriptively lined up dimensionally on distinctive aspects of mental representations, with the AD/D group in the middle between BPD and CON. This pattern of results in part supports both the disorder-specific (EE) and the transdiagnostic (NC, hostile attributions) hypotheses. However, when controlling for education, only the results that supported transdiagnostic associations remained significant.

### Maternal representations in mothers with BPD vs. mothers without mental disorders (CON)

To our knowledge, this is the first study to investigate different aspects of mental representations in mothers with BPD compared with a non-clinical control group. As for the content of these mental representations, we explored criticism and hostility toward the child and their relationship in general (EE) as well as specifically relating to child misbehavior (attribution). Our results are in line with existing evidence that BPD is linked to more criticism [23, 24]. Mothers with BPD are known to be more sensitive to infant anger and report it more often compared with independent observers [85], a result that may be reflected in the EE construct. Our results support an association with increased criticism in mothers with BPD, specifically in covert criticism, while differences in overt criticism seem to be mostly driven by differences in maternal education. Note that the amount of EOI in our sample was too low to be analyzed separately. Our data also supported the hypothesis that BPD is associated with more negative, hostile attributions of infant’s behavior, indicating a bias toward perceiving the child’s (mis-)behavior as intentional. Previous literature indicates that mothers with high BPD characteristics perceive their children as angry compared with ones with low BPD characteristics [86] and tend to blame their child more for misbehavior compared with mothers with depression [71]. Criticism in parents is associated with observational indices of parent-child interactions [36] and also with both internalizing and externalizing problems in youths, thereby rendering it relevant for an intervention [18].

Besides the content of the mental representations, we also included a measure of the structure of the mental representations (NC). Core features of BPD are unstable relationships and a disturbed self-image – both symptoms that may also influence the mental representations that a mother with BPD has not only about herself but also about her child. The literature on the cognitive functioning of patients with BPD indicates that BPD is linked to a disorganized recall of memories with a bias toward negative experiences in comparison with healthy controls [87]. Regarding their experiences with their own parents, mothers with BPD are also less likely to coherently narrate both positive and negative relationship experiences and are more often classified as preoccupied and unresolved in their attachment styles [88]. We identified a similar pattern of results with their own children where mothers with BPD were also less likely to report information with a balanced, consistent, and coherent style.

### The role of maternal education in maternal representations

Regarding criticism and coherence, mothers from the BPD group were predicted to express more covert criticism and less coherence relative to mothers without a current mental disorder in our study, even when maternal education was controlled for. Differences in overt criticism, on the other hand, seem to be more driven by maternal education. Overt criticism in particular (i.e., harsher and more drastic expressions of discontent with one’s own child) should therefore not be regarded as a characteristic of mothers with BPD but rather as a characteristic of mothers with low educational status. Low education status is already known to be a risk factor for more conflicts and less positive relationship quality between parents and their children, most likely due to more (financial) stress and fewer resources [13, 89]. It is crucial to acknowledge that low educational status may also serve as an indicator of other factors that were not measured in our study and that we could not consider for interpretation, such as socio-economic status. Financial stress may be particularly relevant for family distress experiences [90]. The proportion of mothers with a low educational status was very high, particularly in the BPD group, a finding that demonstrates a cumulation of risk factors (here, maternal mental disorder and low educational status combined). Though maternal education also additionally predicted the probability of expressing covert criticism and being incoherent, with higher probabilities for mothers with low educational backgrounds (thereby replicating a well-known finding [91]), BPD group membership still remained a significant predictor. The consistent significant role that maternal education plays in family climate may indicate that education is less of a covariate (i.e., error variance that should be removed from the possible relationship between group and an aspect of mental representations) and instead represents true variance and unfolds its effect most strongly in overt criticism. This idea is supported by the fact that the effects of group membership on the respective aspect of mental representation were always reduced when education was added compared with when it was absent from the models. Furthermore, this effect aligns with models that propose social and contextual influences (e.g., employment or social support) as relevant mechanisms in the intergenerational transmission of BPD [92].

### Maternal representations in mothers with BPD vs. mothers with depression and/or anxiety disorders (AD/D)

As for the content of maternal mental representations and in contrast to our expectations, we did not find significant differences between the two clinical groups in covert criticism and incoherence. Mothers with BPD and AD/D seemed somewhat similar in the extents to which these aspects of mental representations contributed to family climate. Regarding overt criticism, however, mothers with BPD still expressed roughly four times more overt criticism than mothers with AD/D. This difference implies that mothers with BPD have a higher risk for more severe forms of criticism (i.e., overt criticism) than mothers with AD/D. Milder and less drastic forms of criticism in terms of severity (i.e., covert criticism), however, occurred with comparable frequency in the two groups. It is important to note that frequent, less severe criticism may be more damaging to the family climate than infrequent, more severe criticism. At least when it comes to family violence, the chronic presence of milder forms of violence seems to contribute to a more severe violent climate [93]. The distinctive effects of frequency versus severity have not been studied for EE.

Previous literature has already identified an association between depression and anxiety disorders in parents and more criticism [17, 40, 41], yet our results indicate that the expression of criticism is not specific to parents with AD/D but is also present in mothers with BPD. We thus assume that expressing covert criticism may be seen as a transdiagnostic phenomenon that is evident in different mental disorders. However, overt criticism seems to be specifically associated with BPD, but only when maternal education is not controlled for. The disorder-specific hypothesis may thus also be (co-) driven by third factor variables from the social rather than the psychological or biological perspective.

Regarding hostile attributions as a specific aspect of mental representations of child misbehavior, the Jonckheere-Terpstra trend analysis across all groups supported our expectation that mothers with AD/D would be less hostile in their attributions than mothers with BPD, even if the difference was not large enough to reach significant levels across the two-group post hoc comparisons. When examining hostility using behavioral observation, Kluczniok and colleagues [94] demonstrated that mothers with BPD were more inclined to display animosity than mothers with depression. We saw a similar effect in parental attributions, but we cannot conclusively determine whether increased hostility in BPD compared with AD/D can be extended from the level of mental representations to the behavioral level (i.e., to actual dyadic behavior). There is tentative evidence for a trend in this direction, but the differences between the two groups might not be as large as at the behavioral level, so that the two-group post hoc comparisons were not significant. Hostile attributions are a known risk factor for child maltreatment [95] and may thus be an indicator in maltreatment profiles. These attributions tentatively distinguish between parents with different mental disorders and may be an appropriate tool for the early detection of child maltreatment risk in mothers with BPD.

With regard to the organization of mental representations, it should be noted that, on a descriptive level, the majorities of mothers in both clinical groups were coded as incoherent while the majority of mothers in the CON group was coded as coherent, a finding that is to some extent in line with our hypothesis that both clinical groups would have worse family climate than mothers without a current mental disorder. Interestingly, and also contrary to our expectations, the clinical groups were not significantly different from each other in this regard. By investigating autobiographical memories, other researchers discovered a more negative hedonic tone and more reported negative experiences in patients with BPD and patients with depression compared with a healthy control group [96]. Our AD/D sample consisted of roughly 41% mothers with depression, and this large proportion of mothers with depression may have increased the rates of incoherence in the AD/D group. During the coding of narrative coherence, a balance of positive and negative descriptions or challenging aspects in interactions with the child were coded as coherent. The non-significant prediction of incoherence by mental disorder (BPD vs. AD/D) may be explained by shared negativity biases between the two groups: On the one hand, the aforementioned bias of recalling negative experiences in one’s own biography may also have negatively affected the recall of experiences with one’s own child and may have resulted in one-sided narratives. On the other hand, BPD and depression are both associated with a negative perception bias, i.e., a tendency to perceive facial expressions negatively [97, 98], which may also lead to a more negative perception of the child in general and to these mothers’ narratives being coded as incoherent. However, it is also possible that clinical groups do not have a greater negative bias but instead have a lower positive bias than healthy mothers (a finding that was supported by the fact that both clinical groups descriptively showed more incoherence than CON mothers). It is known from community samples that mothers tend to evaluate their own child’s behavior with a positive bias [99]. This tendency thus suggests that mental disorders such as BPD or AD/D lack a positive bias in the perception of child behavior and do not necessarily lead to a stronger perception of negative behaviors. Besides biases in retrieval or perception, incoherence in the FMSS in both clinical groups may be influenced by the mothers’ own attachment-related experiences. As attachment theory proposes, (negative) experiences with one’s own previous attachment figures (referred to as “ghosts in the nursery”) may influence one’s present mental representations [100] and may lead to a “confusion between the relationship with her present child and the relationship to traumatizing or traumatized figures from her past” [101]. Distorted, inconsistent, and incoherent representations of their child are observable in mothers with PTSD [101] as well as in mothers with depression [102, 103]. As individuals with BPD are also known to often have had negative experiences with their caregiver [104, 105], similar mechanisms may apply to this group, leading to incoherent representations of the child in the present.

### Disorder-specific vs. transdiagnostic associations with indicators of maternal mental representations

By including a clinical control group, we aimed to explore whether the potential effects of maternal BPD on mental representations are a disorder-specific or a transdiagnostic phenomenon. Mothers from the BPD group were associated with more overt criticism relative to mothers with depression or anxiety disorders in our study (when maternal education was not controlled for), supporting a disorder-specific association between BPD and this aspect of mental representations. At the same time, our data also suggest that a distinction between different mental disorders may be less relevant across the other aspects of mental representations: Taking a descriptive look, it is striking that the heterogeneity in (covert) criticism, narrative coherence, and hostile attributions increased along group membership, i.e., the largest heterogeneity occurred in the BPD group, followed by the AD/D group, pointing to the potential for identifying high-risk subgroups across disorder group membership instead of focusing on type of disorder for family climate.

### Limitations

Some limitations of this study should be mentioned, namely, that none of the regression models explained a lot of variance (mostly *R*² < 0.15). This trend means that neither group status alone nor the combination with education level contributed greatly to the mental representations we investigated, and other characteristics must be kept in mind. Additionally, at times, the reliability of our coders was below the level of moderate agreement, despite ongoing supervision and discussion of difficult-to-code transcripts. EE1 showed the greatest reliability, suggesting that overt criticism is easier to learn and detect during training. Due to funding constraints and the project timeline, we were unfortunately only able to code 5% of our transcripts for reliability. Any potential methodological issues, such as shared method variance, arising from using the FMSS as a data source for assessing two constructs should also be considered. Additionally, we explored family climate only in terms of different aspects of mental representations in this study. Further studies should investigate whether these maternal representations are also reflected in actual dyadic maternal behavior. While we collected data on dyadic behavior (also including child dyadic behavior), the respective analyses have not yet been completed. However, it seems worthwhile to not only rely on self-report and also approach family climate from a behavioral level because behavioral observation may be less biased by linguistic skills or responding in a socially desirable manner, thereby enabling us to examine parental behaviors that the parents themselves cannot report on [106, 107].

A major limitation is the single-source assessment of the maternal characteristics that contribute to family climate without including information from the child’s perspective, such as their mental representations of the family climate or child agency. As our sample included children between the ages of six months and six years, we refrained from assessing their mental representations, as only the older children would have been able to provide insight into such representations. However, on a behavioral level, we also assessed child-dyadic behavior during two mother-child interaction tasks. These analyses are not yet available but are in the final preparation stages.

Moreover, the issue of potential comorbidities should be considered, and some light should be shed on the AD/D group. We selected this group with a focus on high prevalence rates and therefore chose depressive and anxiety disorders. Due to high comorbidities between depression and anxiety disorders [70], we decided to treat mothers with such disorders as one group. However, there is some evidence that mother-child interactions may differ between mothers with depression and mothers with anxiety disorders. For example, mothers with anxiety disorders have been observed to be more intrusive and to have a heightened synchrony (also compared with healthy controls), whereas mothers with depression seem to be less structured and low in synchrony [12, 108]. In general, it could be argued that mothers with anxiety disorders tend to do “too much” and mothers with depression tend to do “too little”. It is therefore plausible that potential effects on mental representations cancel each other out. This phenomenon may explain why some comparisons between BPD and AD/D were not significant.

Comorbidity in terms of mental disorders besides BPD, depression, and anxiety disorders may also influence mothers’ mental representations. Although the relevant information was collected, controlling for comorbidity is extremely challenging with the various diagnoses that are usually co-morbid with BPD. However, it could potentially impact the outcomes. Similarly, the association between BPD and greater impairments in different aspects of mental representations may also be due to the severity of the impairment [109]. This may also explain the large heterogeneity in the BPD group and should be kept in mind. Lastly, other factors, such as the significant differences in maternal and child age, were not considered in the statistical analyses. Younger children, for example, may misbehave more or less often, which may lead to the expression of more or less criticism and the reporting of more or fewer hostile attributions. As children in the CON group were significantly younger than those in the BPD group, this difference may have impacted the effects in this specific comparison. At the same time, however, it should be noted that, although the age of the children differed significantly, they were within a similar range (average difference of seven to eight months). A meta-analysis revealed that child age is a significant moderator of the relationship between parental mental disorders and EE when considered as a categorical variable [17]. Here, adolescence was a significant moderator, and no statement was made for other age categories [17].

### Implications

First, the results highlight an association between more impaired mental representations and BPD in comparison with mothers without a mental disorder: BPD was associated with more criticism, less coherence, and more hostile attributions. Compared with mothers with AD/D, mothers with BPD did not express more covert criticism and did not show more incoherence. Instead, mothers with BPD expressed more overt criticism and hostile attributions. Additionally, the majority of our sample in either group (BPD or AD/D) was not critical – neither overt nor covert. This information may be beneficial to provide when working with mothers with BPD: These moms are known to have extremely low self-reported parenting confidence [110]. Letting them know that their disorder does not inherently entail a tendency to be overly critical and that other parents with mental disorders also face similar challenges may boost their self-confidence (“it is not only me”) and may motivate them to participate in interventions aimed at improving family climate (e.g., parenting programs). Furthermore, it may be useful to inform service providers across care sectors (e.g., youth welfare, mental health providers) that even a very complex and impairing disorder such as BPD does not necessarily imply that these mothers are not capable of interacting appropriately with their children. This information may help provide a more balanced picture of the strengths and difficulties mothers with BPD may encounter, at least when raising a child between six months and six years of age. On the basis of our data, we suggest that practitioners working with mothers experiencing mental health issues and difficulties with mental representations may address these problems by working specifically with them to reduce the number of critical remarks and the tendency to misinterpret children’s behavior as hostile and intentional.

There are various opportunities to select parenting programs. One promising approach based on the present results – large percentages of incoherent narratives in particular – may be mentalization-based parenting interventions (e.g., the Lighthouse Parenting Program). This intervention may have the potential to enhance mothers’ ability to understand their children’s perspectives [111, 112]. However, more evidence is needed (e.g., randomized controlled trials of this intervention) before referring them to an evidence-based intervention. Another promising approach based on the present results – high EE in particular – may be an intervention that is more embedded in social learning theory, such as Parent-Child Interaction Therapy [113] or Triple P [114]. This type of work seems particularly important because of the heightened risk of the heritability of personality disorders for children of mothers with BPD [115] and the risk for family transmission [92], where even a slightly elevated level of criticism may act as an experience of invalidation through misattribution where a parent communicates that undesirable behaviors are inappropriate or invalid [116], thereby increasing their children’s risk of developing BPD themselves.

Second, more drastic forms of criticism (i.e., overt criticism) seem to differentiate better between different mental disorders than less extreme forms (i.e., covert criticism). For practitioners, this difference may imply that they should pay attention to mothers’ harsh remarks about their children’s behavior and to mothers’ negative attributions of child intentions. Clear rejection of a child’s behavior or characteristics in general, as well as negative attributions about misbehavior in particular, may indicate impaired maternal mental representations. As these representations are assumed to influence concrete dyadic behavior [58], recognizing these characteristics in parents could help practitioners identify an appropriate starting point for interventions.

Third, however, it should be noted that the expression of criticism is also linked to educational status. In particular, more drastic, harsher forms of criticism are confounded with a lower educational status. As mothers in our BPD group in particular often had less education, the results were intertwined there. A lower educational background in itself also predicted covert criticism and incoherent representations, meaning that mothers with lower educational backgrounds are more likely to have one-sided representations of their child, and their children are more likely to grow up in a critical family climate. Practitioners may consider not focusing only on diagnoses of mental disorders but also on the educational backgrounds of their clients, as our findings suggest that disorder membership alone may be a limited source of information for predicting family climate. We recommend that hostile attributions should be considered when working with mothers with BPD.

Finally, we would like to point out that valuable information can be obtained from a mother’s brief description of her child. Gathering information on how and what a parent speaks and thinks about their child is a good way of obtaining information about the parent’s mental representations and family climate. Particularly for mental health professionals, such as psychotherapists or psychiatrists, who primarily work with adults, this can be an easy way to gain information about a challenging mental representation. Working primarily with adults (specifically parents) gives practitioners less insight into family relationships, attitudes, and practices by default. One straightforward method for acquiring insight into parental mental representations is by observing how an adult patient with children characterizes their relationship and their child.

## Conclusion

The present study significantly contributes to a better understanding of the specifics as well as the transdiagnostic associations of maternal BPD and the different aspects of mental representations that contribute to family climate. Compared with mothers with no current mental disorder, BPD was associated with more overt and covert criticism, more incoherence, and more hostile attributions. In comparison with a mixed group of mothers with anxiety and/or depressive disorders, only the association with more overt criticism and more hostile attributions was more strongly linked to BPD. At the same time, low maternal educational status itself was related to more criticism and less coherence, with the BDP-specific result for overt criticism (i.e., harsher and more drastic forms of dissatisfaction with one’s own child) dropping when education was included in the analyses. However, mothers with BPD were predicted to express less coherence relative to mothers without a mental disorder, even after maternal education was controlled for. This finding emphasizes the strong link between a BPD diagnosis and low maternal education and implies that social aspects are not to be neglected when assessing and planning treatment services. Our results also suggest that identifying high-risk groups for persistent difficulties in mental representations across different disorders may be more appropriate. Mothers in this high-risk subgroup and their children may benefit from an orientation toward more family-oriented strategies, including strategies developed to improve mental representations and parent-child relationship quality specifically. When working with mothers who are experiencing mental health issues, specific attention may be directed toward hostile attributions and expressions of criticism in order to identify families in distress. Efforts to reduce maternal critical remarks and to attribute child misbehavior as less intentional may be promising for positive child outcomes.

## Supplementary Information

Supplementary Material 1.

## Data Availability

The scripts used to analyze the present data are available in the OSF repository, https://osf.io/nux7v/ (DOI: 10.17605/OSF.IO/NUX7V). Raw data supporting the conclusions of this article will be uploaded into the repository in completely anonymized form after the publication of all preregistered research questions of the consortium. Until then, the data sets used and/or analyzed during the current study are available from the corresponding author upon reasonable request.

## Abbreviations

AD/D: Anxiety Disorders and/or Depression
BPD: Borderline Personality Disorder
CON: No current Mental Disorder
CV: Child Vignettes
EE: Expressed Emotion
EE1: Expressed Emotion Score 1 (i.e., EE including EOI and overt criticism only)
EE2: Expressed Emotion Score 2 (i.e., EE including EOI, overt and covert criticism)
EOI: Emotional Overinvolvement
FMSS: Five Minute Speech Sample
HEE: High Expressed Emotion
LEE: Low Expressed Emotion
NC: Narrative Coherence
SCID-5: Structural Clinical Interview for DSM-5 Disorders
