# Supplementary material for: Maternal assessments of family climate in mother-child dyads: investigating the role of maternal borderline personality disorder in mental representations
Source: Borderline Personal Disord Emot Dysregul. 2025 Sep 8;12:36. doi: 10.1186/s40479-025-00306-2 (PMC12418619; doi:10.1186/s40479-025-00306-2)
Supplement: Supplementary file 1 — Supplementary Material 1. [file 40479_2025_306_MOESM1_ESM.docx]

**Maternal Assessments of Family Climate in Mother-Child Dyads: Investigating the Role of Maternal Borderline Personality Disorder in Mental Representations – Supplementary Appendix**

## S1: Correlations and Chi²-tests between the independent and dependent variables.

We conducted correlation and chi-square tests between the dependent variables to determine whether the constructs were sufficiently distinct. We anticipated small to medium sized correlations between the dependent variables, assuming the variables cover different constructs.

All outcomes indeed correlated significantly with each other, mostly to a small to medium effect, with the exception of covert criticism and parental attribution which did not correlate significantly.

Table S 1. Correlations with confidence intervals.

| Variable | 1 | 2 | 3 | 4 | 5 |
| --- | --- | --- | --- | --- | --- |
| 1. Group |  |  |  |  |  |
| 2. EE1 | -.15** |  |  |  |  |
|  | [-.26, -.04] |  |  |  |  |
| 3. EE2 | -.19** | .46** |  |  |  |
|  | [-.29, -.08] | [.37, .54] |  |  |  |
| 4. NC | -.33** | .14* | .25** |  |  |
|  | [-.42, -.22] | [.03, .24] | [.15, .35] |  |  |
| 5. CV | -.26** | .04 | .24** | .13* |  |
|  | [-.36, -.15] | [-.07, .15] | [.14, .35] | [.02, .24] |  |
| 6. Education | .42** | -.17** | -.19** | -.25** | -.12* |
|  | [.33, .51] | [-.28, -.07] | [-.29, -.08] | [-.35, -.14] | [-.23, -.01] |
| *Note.* EE1: Expressed Emotion score 1 (overt criticism), EE2: Expressed Emotion score 2 (including covert criticism), NC: Narrative Coherence, CV: Child Vignettes (to assess hostile attributions). Group was included as a factor variable with BPD, ADD and CON as categories (in that order). Between two nominal variables (e.g., EE and NC), we calculated phi coefficients as a measure of correlation. Between an interval scaled variable and a dichotomous variable (e.g., CV and EE), we calculated biserial correlations. Values in square brackets indicate the 95% confidence interval for each correlation. * indicates *p* < .05. ** indicates *p* < .01. | | | | | |

Table S 2. Chi²-Test for EE1 (overt criticism) and EE2 (covert criticism).

| EE1 (overt criticism) | EE2 (covert criticism) | |
| --- | --- | --- |
|  | HEE | LEE |
| HEE | 34 | 0 |
| LEE | 83 | 208 |
| *Note*. N = 325. $\chi^{2}$(1) = 67.51, *p* < .001**, Cramer’s *V* = 0.46 (i.e., medium effect). We used Fisher’s exact test to test the significance due to cells with fewer than five observations. HEE = high expressed emotion, LEE = low expressed emotion. | | |

Table S 3. Chi²-Test for EE1 (overt criticism) and narrative coherence (NC).

| EE1 (overt criticism) | Narrative Coherence | |
| --- | --- | --- |
|  | incoherent | coherent |
| HEE | 24 | 10 |
| LEE | 141 | 150 |
| *Note*. N = 325. $\chi^{2}$(1) = 5.97, *p* = .015*, Cramer’s *V* = 0.14 (i.e., small effect). HEE = high expressed emotion, LEE = low expressed emotion. | | |

Table S 4. Chi²-Test for EE2 (covert criticism) and narrative coherence (NC).

| EE2 (covert criticism) | Narrative Coherence | |
| --- | --- | --- |
|  | incoherent | coherent |
| HEE | 79 | 38 |
| LEE | 86 | 122 |
| *Note*. N = 325. $\chi^{2}$(1) = 20.53, *p* < .001**, Cramer’s *V* = 0.25 (i.e., small effect). HEE = high expressed emotion, LEE = low expressed emotion. | | |
|  | | |

## S2: Testing assumptions for model with group as predictor and EE1 score as outcome

We explored potential outliers and influential cases before running the model. 8 potential outlier were identified (i.e., cases with large standardized residuals (1)). However, since none of these cases had a high influence on the prediction (i.e., Cook’s distance < 1, DFBeta < 1, no conspicuous hat values), we remained from excluding these cases and run the model with the whole sample (1).

Assumption for linearity between predictor and the outcome was given due to the nature of dichotomous (or better dummy-coded) variables. Since we only assessed each outcome only once in each participant, the independence of error was given. Since we only included one predictor in our model, multicollinearity did not apply.

The model fit indicated a good fit: $\chi$^2^(2) = 10.15, *p* = 0.006.

## S3: Testing assumptions for model with group and maternal education as predictors and EE1 score as outcome

To test if including maternal education improves the model fit, we compared the fit of the two models ($\chi$*²_comparison_*(1) = 4.02, *p* = .045), which indicates that the model with education has a better fit. Also, *R*² was higher for the second model (*R*²_with education_ = .065; Hosmer-Lemeshow) than for the first model (*R*²_without education_ = .047; Hosmer-Lemeshow). The AIC for model 1 was also larger than for model 2 (AIC_model1_ = 213.67 > AIC_model2_ = 211.65), indicating a better fit of the model with education included.

We explored potential outliers and influential cases before running the model. 1 potential outlier was identified (i.e., cases with large standardized residuals (1)). However, since this case did not have a high influence on the prediction (i.e., Cook’s distance < 1, DFBeta < 1, no conspicuous hat value), we remained from excluding the case and run the model with the whole sample (1).

Assumption for linearity between predictor and the outcome was given due to the nature of dichotomous (or better dummy-coded) variables. Since we only assessed each outcome only once in each participant, the independence of error was given. We also tested for multicollinearity using VIF and tolerance scores. VIF scores > 10 are concerning and > 1 may indicate a bias (tolerance < .1 and < .2, respectively). Since our scores were below these thresholds (VIF_group_ = 1.18, VIF_education_ = 1.18, tolerance_group_ = 0.85, tolerance_education_ = 0.85), we did not have multicollinearity in our sample.

The model fit indicated a good fit: $\chi$^2^(3) = 14.17, *p* = 0.003.

## S4: Testing assumptions for model with group as predictor and EE2 score as outcome

We explored potential outliers and influential cases before running the model. No potential outlier was identified (i.e., cases with large standardized residuals (1)), and we therefore run the model with the whole sample.

Assumption for linearity between predictor and the outcome was given due to the nature of dichotomous (or better dummy-coded) variables. Since we only assessed each outcome only once in each participant, the independence of error was given. Since we only included one predictor in our model, multicollinearity did not apply.

The model fit indicated a good fit: $\chi$^2^(2) = 12.24, *p* = 0.002.

## S5: Testing assumptions for model with group and maternal education as predictors and EE2 score as outcome

To test if including maternal education improves the model fit, we compared the fit of the two models ($\chi$*²_comparison_*(1) = 4.88, *p* = .027), which indicates that the model with education has a better fit. Also, R² was higher for the second model (*R*²_with education_ = .040; Hosmer-Lemeshow) than for the first model (*R*²_without education_ = .029; Hosmer-Lemeshow). The AIC for model 1 was also larger than for model 2 (AIC_model1_ = 418.49 > AIC_model2_ = 415.6), indicating a better fit of the model with education included.

We explored potential outliers and influential cases before running the model. No potential outlier was identified (i.e., cases with large standardized residuals (1)), and we therefore run the model with the whole sample.

Assumption for linearity between predictor and the outcome was given due to the nature of dichotomous (or better dummy-coded) variables. Since we only assessed each outcome only once in each participant, the independence of error was given. We also tested for multicollinearity using VIF and tolerance scores. VIF scores > 10 are concerning and > 1 may indicate a bias (tolerance < .1 and < .2, respectively). Since our scores were below these thresholds (VIF_group_ = 1.18, VIF_education_ = 1.18, tolerance_group_ = 0.85, tolerance_education_ = 0.85), we did not have multicollinearity in our sample.

The model fit indicated a good fit: $\chi$^2^(3) = 17.12, *p* = 0.001.

## S6: Testing assumptions for model with group as predictor and NC score as outcome

We explored potential outliers and influential cases before running the model. No potential outlier was identified (i.e., cases with large standardized residuals (1)), and we therefore run the model with the whole sample.

Assumption for linearity between predictor and the outcome was given due to the nature of dichotomous (or better dummy-coded) variables. Since we only assessed each outcome only once in each participant, the independence of error was given. Since we only included one predictor in our model, multicollinearity did not apply.

The model fit indicated a good fit: $\chi$^2^(2) = 38.42, *p* < .001.

## S7: Testing assumptions for model with group and maternal education as predictors and NC score as outcome

To test if including maternal education improves the model fit, we compared the fit of the two models ($\chi$*²_comparison_*(1) = 6.31, *p* = .012), which indicates that the model with education has a better fit. Also, R² was higher for the second model (*R*²_with education_ = .099; Hosmer-Lemeshow) than for the first model (*R*²_without education_ = .085; Hosmer-Lemeshow). The AIC for model 1 was also larger than for model 2 (AIC_model1_ = 418.05 > AIC_model2_ = 413.73), indicating a better fit of the model with education included.

We explored potential outliers and influential cases before running the model. No potential outlier was identified (i.e., cases with large standardized residuals (1)), and we therefore run the model with the whole sample.

Assumption for linearity between predictor and the outcome was given due to the nature of dichotomous (or better dummy-coded) variables. Since we only assessed each outcome only once in each participant, the independence of error was given. We also tested for multicollinearity using VIF and tolerance scores. VIF scores > 10 are concerning and > 1 may indicate a bias (tolerance < .1 and < .2, respectively). Since our scores were below these thresholds (VIF_group_ = 1.14, VIF_education_ = 1.14, tolerance_group_ = 0.87, tolerance_education_ = 0.87), we did not have multicollinearity in our sample.

The model fit indicated a good fit: $\chi$^2^(3) = 44.73, *p* < 0.001.

## S8: Testing assumptions for model with group as predictor and hostile attribution as outcome

We explored potential outliers and influential cases before running the model. 16 potential outliers were identified (i.e., cases with large standardized residuals (1)). However, since none of these cases had a high influence on the prediction (i.e., Cook’s distance < 1, DFBeta < 1, no conspicuous hat values), we remained from excluding these cases and run the model with the whole sample (1).

Assumptions for non-zero variance, independence of the outcome values and independent errors were fulfilled; the model had no auto-correlation as the value of the Durbin-Watson statistic was 2.00 (*p* = .916). Since only one predictor was used in the model, multicollinearity did not apply. The assumption of unconstrained data was not fulfilled (outcome intentionally, possible range 6 – 54, data for 6 – 29 only). Additionally, the data did not fulfill the requirements of homoscedasticity and normally distributed errors, as displayed in the following figures (see Figure S1, Figure S2, Figure S3). Due to the number of unfulfilled assumptions, we switched to the Kruskal-Wallis test.

| 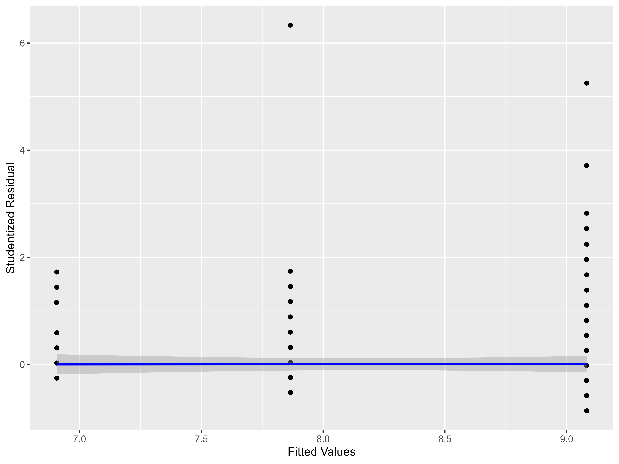  Figure S1. Scatterplot of studentized residuals against predicted values. | 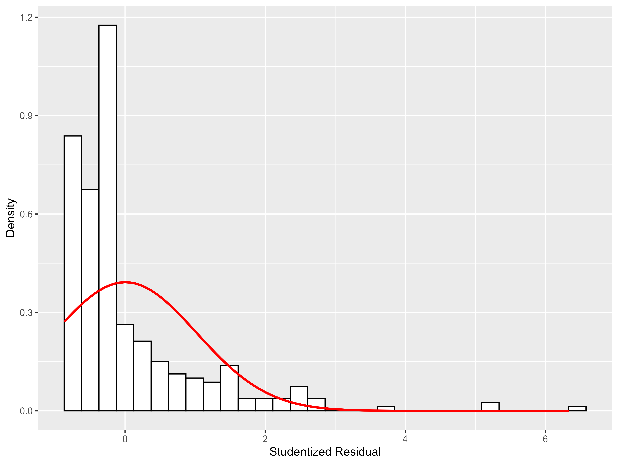  Figure S2. Histogram of studentized residuals. |
| --- | --- |
| 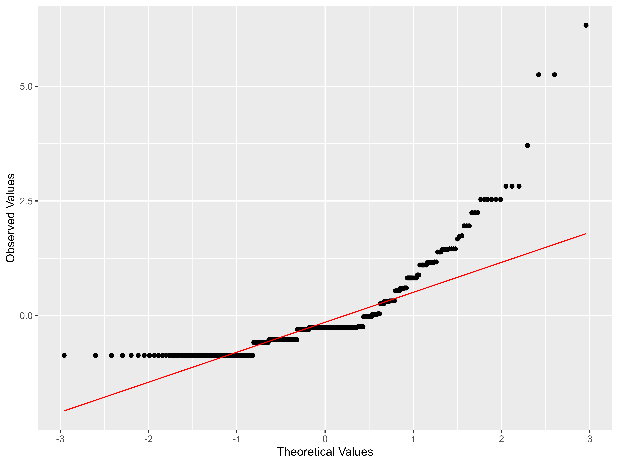  Figure S3. Q-Q plot of predicted and studentized (observed) values. |  |

## S9: Retesting the models without children in foster care

Table S 5. Logistic regression of group (and maternal education) on EE1 score (overt criticism) without children in foster care.

|  | *b* | *SE* | *z* | *p* | Odds Ratio (OR); BPD  vs. CON or AD/D | OR 95% CI;  BPD  vs. CON or AD/D | Odds Ratio (OR); CON or AD/D  vs. BPD | OR 95% CI;  CON or AD/D  vs. BPD |
| --- | --- | --- | --- | --- | --- | --- | --- | --- |
| *Model without Maternal Education* | | | | | | | | |
| Intercept | **-1.72** | **0.22** | **-7.76** | **< .001***** |  |  |  |  |
| Group  (BPD vs. CON) | **-1.113** | **0.51** | **-2.18** | **.029*** | **0.33** | **[0.107 - 0.829]** | **3.04** | **[1.207 - 9.313]** |
| Group  (BPD vs. AD/D) | **-1.309** | **0.63** | **-2.07** | **.038*** | **0.27** | **[0.063 - 0.81]** | **3.70** | **[1.234 - 15.986]** |
| *Model with Maternal Education* | | | | | | | | |
| Intercept | **-2.181** | **0.36** | **-6.07** | **< .001***** |  |  |  |  |
| Group  (BPD vs. CON) | -0.764 | 0.55 | -1.39 | .166 | 0.47 | [0.143 - 1.295] | 2.15 | [0.772 - 6.973] |
| Group  (BPD vs. AD/D) | -1.084 | 0.64 | -1.68 | .093 | 0.34 | [0.077 - 1.051] | 2.96 | [0.951 - 13.003] |
| Education  (high vs. low) | 0.751 | 0.42 | 1.81 | .071 | 2.12 | [0.949 - 4.895] |  |  |
| *Note. N* = 313 (n BPD: 158, n AD/D: 65, n CON: 90), Model without Maternal Education: R² = .044 (Hosmer-Lemeshow), R² = .028 (Cox-Snell), R² = .059 (Nagelkerke). Model $\chi$²(2) = 9.01, *p* = .011*, Model with Maternal Education: R² = .060 (Hosmer-Lemeshow), R² = .039 (Cox-Snell), R² = .080(Nagelkerke). Model $\chi$²(3) = 12.36, *p* = .006**. | | | | | | | | |

Overall, excluding dyads with children in foster care does not produce different results in logistic regressions for overt criticism. The odds ratios (OR) in the model without children in foster care are slightly lower than in the model with children in foster care, but the differences are marginal. However, only maternal education is no longer a significant predictor of expressed emotion.

Table S 6. Logistic regression of group (and maternal education) on EE2 score (covert criticism) without children in foster care.

|  | *b* | *SE* | *z* | *p* | Odds Ratio (OR); BPD  vs. CON or AD/D | OR 95% CI;  BPD  vs. CON or AD/D | Odds Ratio (OR); CON or AD/D  vs. BPD | OR 95% CI;  CON or AD/D  vs. BPD |
| --- | --- | --- | --- | --- | --- | --- | --- | --- |
| *Model without Maternal Education* | | | | | | | | |
| Intercept | -0.229 | 0.16 | -1.43 | .153 |  |  |  |  |
| Group  (BPD vs. CON) | **-0.961** | **0.30** | **-3.24** | **.001**** | **0.38** | **[0.211 - 0.675]** | **2.61** | **[1.481 - 4.75]** |
| Group  (BPD vs. AD/D) | -0.441 | 0.31 | -1.44 | .151 | 0.64 | [0.348 - 1.165] | 1.55 | [0.858 - 2.873] |
| *Model with Maternal Education* | | | | | | | | |
| Intercept | **-0.534** | **0.22** | **-2.42** | **.016*** |  |  |  |  |
| Group  (BPD vs. CON) | **-0.724** | **0.32** | **-2.27** | **.023*** | **0.48** | **[0.256 - 0.898]** | **2.06** | **[1.113 - 3.903]** |
| Group  (BPD vs. AD/D) | -0.278 | 0.32 | -0.87 | .383 | 0.76 | [0.401 - 1.408] | 1.32 | [0.71 - 2.493] |
| Education  (high vs. low) | **0.547** | **0.27** | **2.06** | **.040*** | **1.73** | **[1.026 - 2.913]** |  |  |
| *Note. N* = 313 (n BPD: 158, n AD/D: 65, n CON: 90), Model without Maternal Education: R² = .028 (Hosmer-Lemeshow), R² = .036 (Cox-Snell), R² = .049 (Nagelkerke). Model $\chi$²(2) = 11.44, *p* = .003**, Model with Maternal Education: R² = .038 (Hosmer-Lemeshow), R² = .049 (Cox-Snell), R² = .067 (Nagelkerke). Model $\chi$²(3) = 15.66, *p* = .001**. | | | | | | | | |

Overall, excluding dyads with children in foster care does not produce different results in logistic regressions for covert criticism.

Table S 7. Logistic regression of group (and maternal education) on narrative coherence (NC) without children in foster care.

|  | *b* | *SE* | *z* | *p* | Odds Ratio (OR); BPD  vs. CON or AD/D | OR 95% CI;  BPD  vs. CON or AD/D | Odds Ratio (OR); CON or AD/D  vs. BPD | OR 95% CI;  CON or AD/D  vs. BPD |
| --- | --- | --- | --- | --- | --- | --- | --- | --- |
| *Model without Maternal Education* | | | | | | | | |
| Intercept | **0.572** | **0.17** | **3.45** | **.001**** |  |  |  |  |
| Group  (BPD vs. CON) | **-1.701** | **0.30** | **-5.75** | **< .001***** | **0.18** | **[0.101 - 0.322]** | **5.48** | **[3.108 - 9.95]** |
| Group  (BPD vs. AD/D) | -0.356 | 0.30 | -1.19 | .235 | 0.70 | [0.389 - 1.264] | 1.43 | [0.129 - 0.512] |
| *Model with Maternal Education* | | | | | | | | |
| Intercept | 0.222 | 0.22 | 1.02 | .306 |  |  |  |  |
| Group  (BPD vs. CON) | **-1.435** | **0.31** | **-4.57** | **< .001***** | **0.24** | **[0.127 - 0.436]** | **4.20** | **[2.292 - 7.885]** |
| Group  (BPD vs. AD/D) | -0.162 | 0.31 | -0.52 | .603 | 0.85 | [0.462 - 1.576] | 1.18 | [0.635 - 2.165] |
| Education  (high vs. low) | **0.662** | **0.27** | **2.44** | **.015*** | **1.94** | **[1.142 - 3.312]** |  |  |
| *Note. N* = 313 (n BPD: 158, n AD/D: 65, n CON: 90), Model without Maternal Education: R² = .087 (Hosmer-Lemeshow), R² = .114 (Cox-Snell), R² = .152 (Nagelkerke). Model $\chi$²(2) = 36.75, *p* < .001**, Model with Maternal Education: R² = .101 (Hosmer-Lemeshow), R² = .130 (Cox-Snell), R² = .174 (Nagelkerke). Model $\chi$²(3) = 43.76, *p* < .001**. | | | | | | | | |

Overall, excluding dyads with children in foster care does not produce different results in logistic regressions for narrative coherence.

**Hostile attribution**

Excluding children in foster care resulted in comparable test statistics: The number of hostile attributions differed between groups, *H*(2) = 22.08, *p* < .001. Two-group post hoc comparisons of the mean ranks between the groups revealed a significant difference between the BPD and CON group regarding hostile attributions (*difference* = 50.368, *critical difference* = 28.136), but not between the BPD and AD/D (*difference* = 23.375, *critical difference* = 31.865) or the AD/D and CON group (*difference* = 26.993, *critical difference* = 34.541). As expected, Jonckheere-Terpstra’s test showed a significant trend in the data: more hostile attributions in M-BPD than in M-AD/D, which in turn displayed more hostile attributions than mothers in the CON group, *J* = 11486, *p* < .001.

**References**

1. Field A, Miles J, Field Z. Discovering statistics using R. Repr. Los Angeles, CA, USA: Sage; 2012. 957 p.
